# Design and 3D printing of pelvis phantoms for cementoplasty

**DOI:** 10.1002/mp.17560

**Published:** 2024-12-17

**Authors:** Cléa Sieffert, Laurence Meylheuc, Bernard Bayle, Julien Garnon

**Affiliations:** ^1^ ICube Laboratory University of Strasbourg UMR 7357 CNRS Strasbourg France; ^2^ INSA of Strasbourg Strasbourg France; ^3^ Department of Interventional Radiology University Hospital Strasbourg France

**Keywords:** cementoplasty, interventional radiology, phantoms

## Abstract

**Background:**

Percutaneous image‐guided cementoplasty is a medical procedure for strengthening bones structurally altered by disease, such as osteolytic metastasis. This procedure involves injecting biocompatible liquid bone cement, through one or more trocars into the damaged bone. Within a few minutes the bone cement hardens and restores the rigidity of the bony structure. The introduction of this technique in the case of large cancellous bones, such as the pelvis, raises some practical issues such as: how to manage the flow of cement with variable viscosity over time and how to inject a large amount of cement under fluoroscopy to effectively restore the patient's ability to bear weight?

**Purpose:**

As a means of training for young practitioners to ensure maximal filling of a metastatic bone area, we have designed and manufactured a pelvic phantom capable of replicating cement diffusion in healthy and metastatic bone under fluoroscopic and computed tomography guidance.

**Methods:**

The preliminary stage of the study consisted of an analysis of various lattice structures, with the objective of reproducing the haptic feedback experienced during the needle insertion and diffusion of cement within the trabecular bone. Cementoplasty tests were conducted by an experienced radiologist under fluoroscopy and CT guidance to evaluate the performance of the lattice structure. The initial analysis provided the groundwork for the design of the phantom pelvis, which was then evaluated against a patient case. The phantom was divided into two distinct components: a disposable section with lattice structure, intended for the injection of cement, and a reusable part representing the pelvic bones. Two additive manufacturing methods were selected for the production of the phantom: Stereolithography (SLA) for the lattice structure and Fused Deposition Modeling (FDM) for the pelvic bones. The disposable component was composed of different lattice structures, selected to best match the anatomic conditions of both healthy and diseased areas visible on the patient images. Subsequently, the performance of the phantom was validated against patient images through a cementoplasty test.

**Results:**

A total of 12 distinct lattice structures were subjected to three tests of cementoplasty. Stochastic lattices with 500 microns beam thickness and densities varying from 15% to 5% demonstrated the most effective replication of the needle haptic feedback, as well as the diffusion of the cement into healthy and osteolytic cancellous bone. These structures were then implanted in the phantom and validated against one patient case.

**Conclusions:**

A methodology to design and manufacture a phantom dedicated to cementoplasty from patient images is proposed. Initially, a series of lattice structures, exhibiting diverse structure types, thicknesses, and densities, were evaluated to assess their capacity to accurately reproduce the haptic feedback of the needle and the diffusion of cement in the trabecular bone. Subsequent to the outcomes of these investigations, several structures were selected for the development of a phantom capable of accurately replicating a cementoplasty procedure under fluoroscopy and CT guidance. This phantom will enable the training of future practitioners on the procedure of cementoplasty in the pelvis.

## 1 INTRODUCTION

### 1.1 Cementoplasty of the pelvic bone

Percutaneous cementoplasty is a percutaneous image‐guided intervention, which consists of injecting an acrylic bone cement into the bone through a dedicated bone trocar under fluoroscopic and 3D imaging. This minimally invasive technique has proven to be effective and safe to alleviate the pain related to some osteoporotic fractures or bone metastases.^1^, ^2^, ^3^ Initially developed for the spine, cementoplasty is increasingly being performed in extra‐spinal anatomical sites in cancer patients, notably the pelvis that is the second most frequent site of bone metastases after the spine.^4^ In addition to pain palliation, cementoplasty can be used to stabilize impending/pathological fractures, either as a stand‐alone technique or in combination with screws^5^, ^6^ depending on the size and localization of the target tumor within the pelvic bone. In such case, the goal of the intervention is to enable the patient to weight bear rapidly without the need for an invasive open surgical procedure.

Preclinical studies indicate that load transfer can be restored, provided that the osteolytic areas in the pelvic bone are filled completely with bone cement. Such optimal filling is however hardly achieved in the clinical practice in which the filling rates range between 30% and 60%.^7^, ^8^ The number of bone trocar(s) as well as its (their) positioning, and the flow rate of cement injection may influence the diffusion of the auto‐polymerizing PMMA cement thereby leading to variable volumes and occurrence of cement leakages for a given situation. The formation of trainees is therefore key to ensure maximal filling of a pelvic osteolytic area as this may translate to a better and more long‐lasting clinical result. In this perspective, the development of dedicated pelvic bone models mimicking pathological situations is of interest.

### 1.2 Pelvic phantoms

Educational phantoms are used to improve the understanding and the realization of a procedure by young physicians before they come to the real clinical practice. Often produced from polymer material, phantoms enable easier and more repeatable clinical^9^ or mechanical testing. The recent advances in three‐dimensional (3D) printing technology have enabled the production of new phantoms generated from patient data,^10^, ^11^, ^12^, ^13^, ^14^, ^15^ capable of reproducing a range of pathological conditions^10^, ^11^, ^12^, ^13^, ^15^, ^16^ and providing realistic haptic feedback.^10^, ^13^, ^17^

Pre‐clinical training and testing of injection of acrylic cement into bone represent a specific challenge in which bone models may be of help. Human cadaveric specimens are perfect anatomical models but without the possibility to easily simulate and reproduce a pathological situation. Other limitations of cadaveric models include their limited availability^18^, ^19^, ^20^ and the need to perform the intervention in a dedicated anatomical department, which conditions differ from an interventional suite^18^, ^19^, ^20^ (different room temperatures, low‐quality x‐ray C‐arms). Because of cost and mostly ethical concerns, animals should no longer be used for teaching purposes. Moreover, even large animals like pigs do not represent valuable anatomical models. It also does not offer the possibility to replicate a pathological situation.

Currently available bone phantoms may be interesting for anatomical purposes but do not represent realistic models for cementoplasty. They are generally made of foam, which is not adapted to the diffusion of bone cement, even when they are filled with open foam cells. Given these limitations, hands‐on training of young practitioners to cementoplasty, biomechanical evaluation and preclinical testing of various constructs (cement injection combined or not with screw insertion for example) are limited with current models (Table 1).

**TABLE 1:** Characteristics of commercial and research pelvis and spine phantoms.

Brand or reference	Anatomical site	Pathology target	Manufacturing process	Cementoplasty compatibility
Sawbones	L5 Vertebra	None	Foaming	Yes
	Pelvis	None		No
	Femoral head	Osteoporotic, screw insertion		Yes
3D Scientific	Pelvis	None	Foaming	No
Creaplast	Pelvis	None	Foaming	No
Stefan et al.^10^	Spine	Osteoporotic, trocar insertion	Fused deposition modeling (FDM)	Not specified
Burkhard et al.^19^	Spine	Osteoporotic, screw insertion	MultiJet printing	Not specified
Bohl et al.^32^	Spine	None, screw insertion	FDM and silicon moulding	Not specified
Li et al.^33^	Spine	Spondylosis, facet joint injections	PolyJet, ceramic printing and gelatin moulding	Not specified

The objective of this work is to present our development of a dedicated pelvic bone phantom for cementoplasty with three prerequisites: realistic haptic feedback during needle insertion, reproduction of cement diffusion under fluoroscopy and CT‐scan in cancellous and pathological bones, and a lower cost compared to current models.

## 2 MATERIAL AND METHODS

The material and methods section is structured as follows: Section 2.1 provides an overview of the phantom design. Section 2.2 assesses the capacity of various lattice structures to replicate a cementoplasty procedure. In the final Section 2.3, the phantom pelvis is evaluated through a comparative analysis with a patient case. To conduct this analysis, the structures identified in Section 2.2 as having the most promising results were employed in the design of the phantom. For the study, the data from the CT‐scan of one patient were retrospectively selected and extracted from the PACS of the institution. The patient gave informed consent for the procedure and the use of his data during the pre‐interventional consultation. Given the very small sample of patient and the retrospective analysis of imaging data only, no further institutional board approval was required by the institution.

### 2.1 Phantom design overview

**FIGURE 1:**
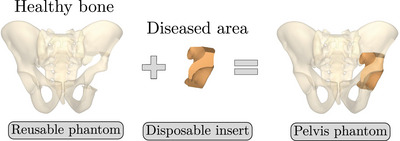
Pelvis phantom splitting approach.

The phantom design was generated by reconstructing CT scan images of a patient with a metastasis in the hip joint region. The pelvic girdle, including the sacrum and two hip bones, was segmented using 3DSlicer ^21^ (version 5.2.2) to generate a surface mesh in stereolithography (SLA) format. The mesh was then cleaned and solidified using Creo ^22^ (version 8.0.4.0) to obtain 3D volumes. Since bone metastases are typically localized in limited areas of the pelvis, the phantom was divided into two parts: a reusable component representing the healthy region of the pelvis and a disposable component representing the diseased region (Figure 1). In order to reproduce the intraosseous path employed fort the insertion of cementoplasty needles into the pelvis, a series of conical openings have been added to the reusable component of the phantom. These holes allow a needle access to the insert along an intraosseous access with a variable angle of $\pm 5^{\circ }$.

Three‐dimensional printing is an appropriate process for the fabrication of complex structures, such as patient‐specific anatomical models. One of the most well‐known processes is fused deposition modeling (FDM) in which a thermoplastic filament is layered through an extrusion nozzle. This technology allows the printing of a wide range of materials at a low cost,^23^ and is particularly suited for large‐volume parts. Consequently, this process has been selected for the production of the reusable component in polylactic acid (PLA) on a Prusa MK3.

### 2.2 Disposable insert representing the diseased area

Although CT scans provide an accurate representation of the boundaries of the patient's bones, they lack the requisite precision to capture the internal bony structure in sufficient detail. It is therefore necessary to design a specific bone model that is capable of accurately replicating cement diffusion in bones, particularly in the context of metastasis. For the treatment of these osteolytic lesions with cementoplasty, the objective of the practitioner is to inject the maximum amount of cement into the affected area (while preventing leakage) and secure the cement within the healthy bone.^2^, ^24^ Consequently, the insert must represent both the diseased and healthy bone in order to reflect the complete cementoplasty procedure, including the interdigitation between the cement and the healthy bone.

At the macromolecular scale, bone is composed of two principal structures: the cortical bone (outer shell) and the cancellous bone (spongy structure). Lattice structures are particularly well‐suited for the reproduction of this architecture. In comparison to foams, they offer the advantage of providing control over both global and local morphology of the structure. Such control simplifies the production of anatomical geometries and allows localized adaptation to different patients and pathologies. The most commonly reported lattice structures used for bone tissue engineering in the medical field are detailed in Table 2. Giroid, diamond and primitive triply periodic minimal surface (TPMS) structures have been extensively employed as bone tissue for implants or prostheses^25^, ^26^ and more recently, for surgical phantoms.^19^ The investigation of truss structures, and more recently stochastic truss, as potential bone substitutes for orthopedic applications appears to be a growing area of interest.^26^, ^27^, ^28^ Stochastic truss, in particular, exhibits a structure that closely mimics that of cancellous bone,^26^, ^28^ thus offering a promising option for our application.

**TABLE 2:** Types of investigated lattice structures.

Type	TPMS			Truss	
Name	Giroid	Diamond	Primitive	Arranged	Stochastic
3D Shape	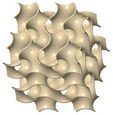	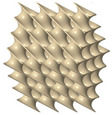	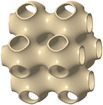	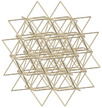	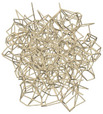

Abbreviation: TPMS, triply periodic minimal surface.

**FIGURE 2:**
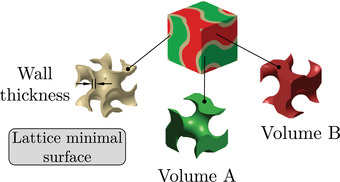
TPMS lattice generated from a minimal surface, which separates the elementary cell in two volumes A and B. TPMS, triply periodic minimal surface.

**FIGURE 3:**
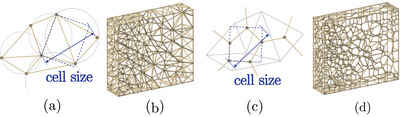
(a) Delaunay triangulation algorithm in 2D with cell size parametrization. (b) Lattice generated with the Delaunay triangulation. (c) Voronoi diagram algorithm in 2D with cell size parametrization. (d) Lattice generated with Voronoi diagram.

Lattice structures are typically composed of an elementary cell, which is then repeated periodically in space to generate the complete structure. The elementary cell of TPMS lattices is generated from a surface defined by a function in space. This surface is then thickened in order to separate the elementary cell into two disjoint volumes, as illustrated in Figure 2. The elementary cell of the second type of lattices considered in Table 2 – the truss type – is an assembly of beams in space. The spatial distribution of the beams can be designed using a stochastic approach. In this case, the size of the cell is determined, and an algorithm generates the lattices from a cloud of points in space. Two generation algorithms are considered and illustrated in Figure 3: (i) the Delaunay triangulation creates triangles from a cloud of points such as the circumscribed circle of a triangle does not contain any other point; (ii) the Voronoi diagram can be obtained from Delaunay's triangulation. The vertices are generated by the centers of the circumscribed circles of the triangles and the edges by the medians of the triangles. Creo computer‐aided design (CAD) software has the ability to generate TPMS or truss lattice structures with two distinct methods. For TPMS lattice, the user defines the wall thickness and dimensions of the elementary cells. For truss lattice, the user defines the beam diameter and dimensions of the elementary cell.

As previously stated, one of the objective is to mimic osteolytic metastases. This pathology alter the structure of bones and consequently the flow of orthopedic cement. Osteolytic metastases lead to modifications in the trabecular bone structure: trabeculas become thinner,^29^ the bone network loses connectivity^29^ and the volume of bone over the total volume (BV/TV) decreases^30^ resulting in a porosity increase of the cancellous bone.

The following subsections analyze three properties of the lattice structures to identify the one that provides realistic haptic feedback during needle insertion, as well as realistic cement diffusion under fluoroscopy and CT scan. The studied parameters include the type of lattice structure, the lattice thickness (wall or beam diameter), and the lattice density. To produce the lattice structures, an alternative additive manufacturing process, called SLA, was selected for its ability to manufacture detailed and complex structures. SLA consists in the selective polymerization of a photosensitive resin using UV light. It is a mono‐material process, more expensive than FDM^23^ and which requires post processing of the printed parts using isopropanol bath and post‐curing with UV light. All lattice structures were printed on a SLA printer Prusa SL1S in Prusament Tough resin.

#### 2.2.1 Lattice type

The first studied property was the type of lattice structure. The objective of this sub‐study was to identify the type of lattice structure that most accurately replicates the injection of cement into bone (healthy or metastatic).

Five types of lattice structure were compared: giroid, diamond, primitive, arranged truss and stochastic truss. A simplified model, consisting of a 30 × 30 × 30 mm cube, was created to test each structure types. In order to isolate the influence of the lattice type, the thickness and the density of the structures were fixed. Based on the capabilities of the production method, the minimum printable thickness and cell size (which define the density of the structure) was determined. Consequently, all samples were manufactured with a beam diameter or wall thickness of 400 microns and a cell size of 5 mm.

Cementoplasty injection tests were performed on each sample to assess the ability of the lattice type to replicate the cement diffusion in bone. All tests were conducted by an experienced interventional radiologist using the following steps:

1. Placement of the sample in the imaging device and insertion of a 10‐gauge beveled needle (Vertebroplasty Needle Optimed) into the sample under fluoroscopy and CT guidance.
2. Preparation of the auto‐polymerizing and radiopaque PMMA cement compound (Osteopal V, Heraeus Medical) and coupling of the manual injection device for cementoplasty.
3. Injection of the cement into the sample under fluoroscopic control.

In order to evaluate the ability of the structure to reproduce the cementoplasty procedure, three criteria have been selected. The first criterion is the volume of cement injected, which was quantified by segmenting the cement from the CT scans of the samples. The second criterion is the overall diffusion pattern through the lattice structure, evaluated by measuring the dimensions of the cylinder circumscribing the volume of cement along the injection axis. The final criterion is a likert scale, which was employed for evaluating the structure that most accurately reproduces the cement diffusion and haptic feedback during needle insertion in bone, based on the assessment provided by the radiologist. Furthermore, the quality of the printed lattice structures was assessed by computing the relative weight difference between the theoretical weight of the CAD model and the actual weight of the printed sample. All samples were tested three times with a 5 mL injection target, and all injections were captured using a system combining a CT scan and a C‐arm (Alphenix, Canon Medical Systems, 120 kVp, Bone kernel, pixel spacing 0.316 mm).

#### 2.2.2 Lattice thickness

Subsequent to the selection of the optimal lattice structure type from the tests conducted in the previous section (results available in Section 3.1.1), the second investigated property was the thickness of the lattice structures. Similarly to the preceding section, the objective was to identify the structure that most closely reproduces the distribution of cement and haptic feedback during needle insertion in bone.

The same simplified cubic model was selected for the tests. The stochastic lattice structures were selected based on prior results of Section 2.2.1. The lattice cell size was fixed at 5 mm, and a range of lattice strut thicknesses, including 400, 500, 600, 700, and 800 μm, was tested.

The same protocol and measurements outlined in previous section were followed to determine the optimal beam thickness for the selected objectives.

#### 2.2.3 Lattice density

Following the selection of the lattice structure type and strut thickness, the final evaluated property was the influence of structure density on haptic feedback during needle insertion and reproduction of cement distribution in the healthy and diseased bone.

The cubic model described previously was selected and filled with stochastic lattices with a thickness of 500 μm, based on experimental results from the previous section (available in Section 3.1.2). A range of structure densities was tested, including 5%, 10%, and 15%. The same protocols and measurements described in previous section were employed to determine the optimal density required to achieve the stated objectives.

### 2.3 Patient‐specific case

The aim of this section is to evaluate the properties of the lattice structures selected to mimic healthy and diseased bone, based on the results of Section 2.2, by comparing them with a clinical case. This was achieved by comparing the injection procedures performed on the phantom model with those conducted on a real patient. Furthermore, this study also aims to analyze the costs associated to the production of the phantom, thus enabling a comparison between the economic impact of our solution and existing alternatives.

The selected patient data in this study are identical to those described in Section 2.1. The patient was diagnosed with an osteolytic metastasis and underwent a cementoplasty procedure, consisting in the injection of 14.8 mL of cement. A comprehensive data set was available for the procedure, including fluoroscopic and CT scan images acquired before, during, and after the operation. Specific details were provided regarding the volume of cement injected into the metastasis and the adjacent healthy bone, in accordance with clinical practice. Additionally, images captured the initial angle and depth of needle insertion, as well as the number of needle withdrawals during the injection process.

#### 2.3.1 Patient‐specific phantom

The design and the production of the reusable component of the phantom, representing the healthy part of the bone, were described in detail in Section 2.1 (see Figure 1).

**FIGURE 4:**
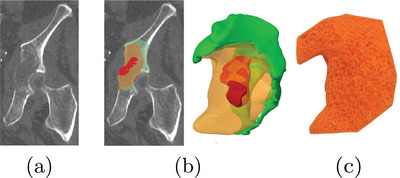
(a) Computed tomography slice of the patient's diseased wing. (b) Different areas identified on the patient's images and the CAD volume of the insert. The green area indicates the healthy trabecular bone, the orange color indicates the area with partial osteolysis secondary to metastasis, and the red color indicates the area with complete osteolysis by the metastasis. (c) Front view of the 3D‐printed insert. CAD, computer‐aided design.

The initial volume of the insert, representing the diseased region of the pelvis, was reconstructed by segmentation of the patient's CT scan. With the practitioner's expertise, both metastatic and healthy bone volume containing the cement were delineated. The two aforementioned volumes were subsequently combined in order to generate the final volume of the insert. Furthermore, a region of complete osteolysis by the metastasis was also extracted from the patient CT scan data. The final volume of the insert was displayed in Figure 4 and contained a volume of 27.4 mL of healthy cancellous bone, 53.6 mL of metastatic bone with partial osteolysis, and 5.9 mL of metastatic bone with complete osteolysis.

In accordance with the results of Section 2.2, a lattice structure with variable properties was assigned to each volume:

1. In the healthy cancellous bone volume: stochastic lattices with a strut thickness of 500  μm and 15% porosity.
2. In the bone volume with partial osteolysis secondary to metastasis: stochastic lattices with a strut thickness of 500 μm and 10% porosity.
3. A void was created in the area with complete osteolysis by the metastasis identified by the practitioner.

The insert was produced using the same method as the other lattice structures described in Section 2.2, namely on a Prusa SL1S SLA printer with the Prusament Tough resin.

#### 2.3.2 Patient‐specific phantom evaluation

**FIGURE 5:**
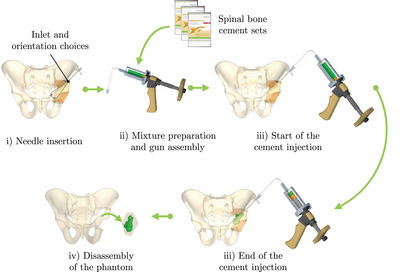
Main steps of the cementoplasty procedure on a pelvis phantom.

The capacity of the phantom to reproduce the distribution of cement within a metastasis was assessed through cement injection, applying the same methodology delineated in Section 2.2.1. In this test, the practitioner responsible for conducting the procedure differed from the one who originally performed the cementoplasty to the patient. However, data specific to the patient, including the angle of insertion, depth of penetration, and the number of needle withdrawals during the original intervention, were employed to guide the practitioner's actions during cementoplasty procedure of the phantom. During the needle insertion phase into the phantom (Figure 5) this data were employed to enhance needle orientation and insertion. The targeted amount of cement injected was identical to that of the operated patient, i.e. 14.8 mL of cement.

The metrics employed to assess the performance of the pelvic phantom are consistent with those adopted for evaluating the lattice structures. They include a comparison of the circumscribed cylinder of the cement injected into the phantom with that of the patient, as well as an assessment of the quantity of cement injected. The insert designed for the phantom included both healthy and metastatic bone, necessitating the implementation of an additional measurement to compare the cement distribution in the phantom with that of the patient. Based on the practitioner's expertise and the CAD model of the insert, the cement in the patient and the phantom were divided into two distinct parts: the cement injected into the healthy bone and the cement injected into the metastatic bone. The quality of the insert was evaluated by computing the difference between the theoretical weight from the CAD model with the actual weight of the produced insert. Based on four produced inserts, mean and standard deviation were calculated. Finally, a comprehensive cost analysis of the phantom was conducted. This analysis considered the costs of materials, machines, and labor, while excluding the costs associated with depreciation and development time.

## 3 RESULTS

### 3.1 Disposable insert

The test results are summarized in the following tables: the type of lattice structure in Table 3, the thickness in Table 4, and the density in Table 5.

**TABLE 3:** Cement injection results for the lattice structure type.

	Giroid	Diamond	Primitive	Stochastic truss	Arranged truss
Manufactured lattice structure	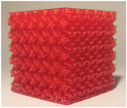	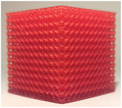	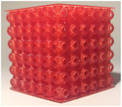	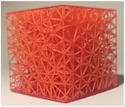	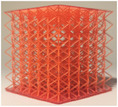
Fluoroscopy image	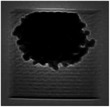	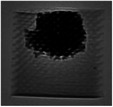	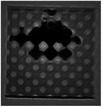	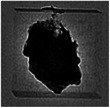	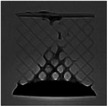
CT scan slice	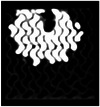	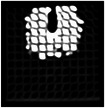	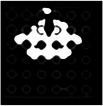	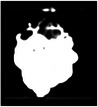	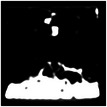
**Cement quantity (mL)**					
Mean	4.1	3.0	3.5	4.7	3.3
Standard deviation	2.1	2.4	3.1	0.8	0.5
**Cylinder dimensions (mm)**					
Mean height	25.3	17.3	20.2	25.5	15.3
Mean diameter	31.5	29.5	31.7	24.9	37.4
**Likert scale [0, 1, 2] 0 = Totally unrealistic 1 = Reminds of reality 2 = Totally realistic**					
Cement diffusion	1	1	0	2	0
Needle insertion	0	1	0	1	0
**Weight difference (%)**					
Mean	14.6	16.7	15.2	4.5	4.5
Standard deviation	2.0	1.5	0.5	0.5	1.0

**TABLE 4:** Cement injection results for different thicknesses of stochastic lattice.

	400 μm	500 μm	600 μm	700 μm	800 μm
Manufactured lattice structure	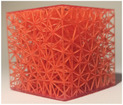	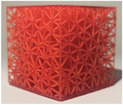	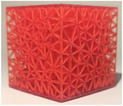	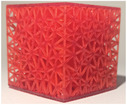	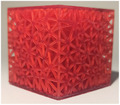
Fluoroscopy image	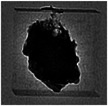	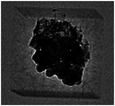	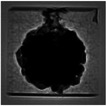	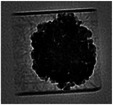	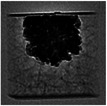
CT scan slice	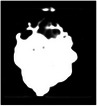	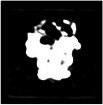	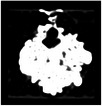	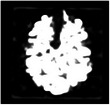	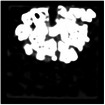
**Cement quantity (mL)**					
Mean	4.7	4.2	4.2	6.5	5.3
Standard deviation	0.8	0.2	0.2	0.8	0.3
**Cylinder dimensions (mm)**					
Mean height	25.5	28.3	26.3	23.5	16.4
Mean diameter	24.9	25.9	25.0	32.2	18.7
**Likert scale [0, 1, 2] 0 = Totally unrealistic 1 = Reminds of reality 2 = Totally realistic**					
Cement diffusion	2	2	2	2	1
Needle insertion	1	2	2	1	1
**Weight difference (%)**					
Mean	4.5	1.0	4.0	1.6	2.5
Standard deviation	0.5	0.5	1.5	0.8	0.9

**TABLE 5:** Cement injection results for different density of stochastic lattice.

	5%	10%	15%
Manufactured lattice structure	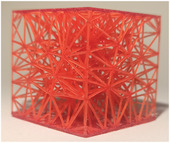	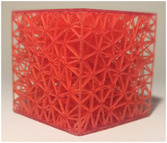	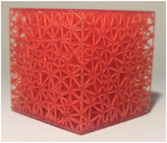
Fluoroscopy image	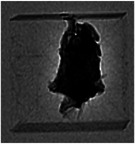	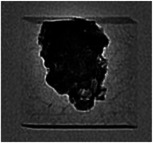	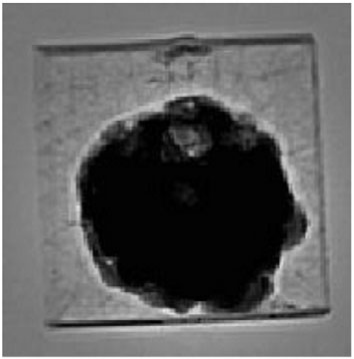
CT scan slice	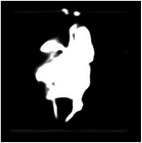	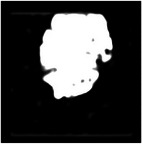	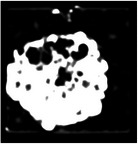
**Cement quantity (mL)**			
Mean	3.2	4.4	6.0
Standard deviation	0.7	0.3	0.7
**Cylinder dimensions (mm)**			
Mean height	25.9	28.2	25.6
Mean diameter	25.4	24.3	31.3
**Likert scale [0, 1, 2] 0 = Totally unrealistic 1 = Reminds of reality 2 = Totally realistic**			
Cement diffusion	1	1	2
Needle insertion	0	1	2
**Weight difference (%)**			
Mean	2.5	1.7	3.1
Standard deviation	0.6	0.6	1.1

Each table includes the following data:

1. a photograph of the manufactured structure (row no. 1);
2. an X‐ray image captured at the end of the injection, taken perpendicularly to the injection axis (row no. 2);
3. a scan section taken at the end of the injection, in the plane that passes through the injection axis (row no. 3);
4. the mean and standard deviation of the cement volume for each sample (row no. 4);
5. the mean dimensions (height and diameter) of the circumscribed cylinders of the injected cement (row no. 5);
6. a likert scale for cement diffusion and needle insertion, rating the structure from most realistic to least realistic (row no. 6);
7. the mean and standard deviation of the weight difference between the CAD and the produced structure (row no. 7).

In order to establish a comparison for the images obtained during the tests on the lattice structures, three patient cases are presented in Table 6. All patients suffered from traumatic vertebral fractures and were treated with cement injections. The cases were selected for their concordance in the quantity of cement injected into the cubes and the vertebrae. Furthermore, as these patients suffered from non‐pathological fractures, the diffusion of cement in the trabecular bone was representative of that in healthy cancellous bone, thus providing a reference for the cement distribution presented in the results.

**TABLE 6:** Patient data with fluoroscopy and CT images of cement injection in nonpathological vertebrae.

	Patient case no. 1	Patient case no. 2	Patient case no. 3
Fluoroscopy image	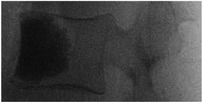	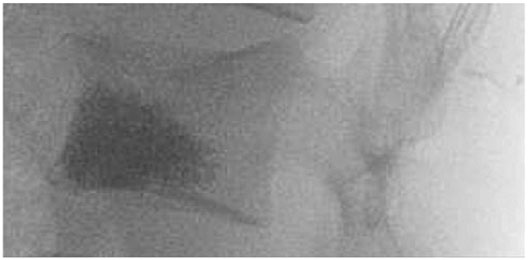	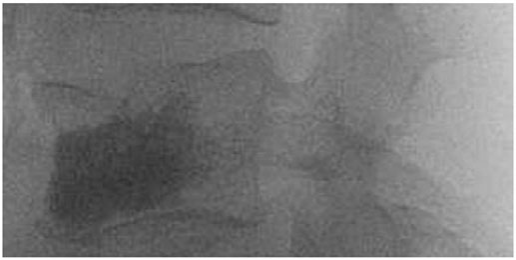
CT scan slice	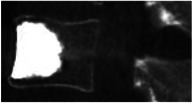	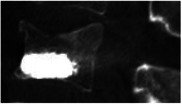	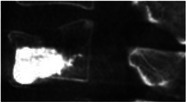
Cement quantity	4.0 mL	3.0 mL	3.5 mL

#### 3.1.1 Lattice type

In the test series comparing different types of lattice structure, the maximum quantity of injected cement was 5.7 mL, while the minimum quantity was 1.3 mL. Consequently, the maximum height of the cylinder was 29.6 mm, with a minimum of 11.2 mm. Similarly, the maximum diameter of the cylinder was 41.3 mm, with a minimum of 24.5 mm.

In the evaluation of the haptic feedback during needle insertion, two structures were found to be of interest: the diamond structure, which exhibited a higher rigidity than healthy bone, and the stochastic structure, which exhibited a lower rigidity than healthy bone according to the radiologist. The evaluation of cement diffusion revealed that the stochastic lattices were of great interest to the practitioner. The giroid and diamond structures were also interesting from a fluoroscopic perspective. However, it was pointed out by the practitioners that all TPMS structures exhibited a too‐oriented diffusion of cement, as evidenced by the CT slices presented in the Table 3. The arranged lattice structure demonstrated poor cement retention, with the cement dropping to the bottom of the cube. Lastly, the TPMS structures revealed a higher weight difference than the truss structures.

#### 3.1.2 Lattice thickness

In the series of tests varying the lattice thickness, the maximum quantity of injected cement was 7.0 mL, while the minimum quantity was 4.1 mL. Subsequently, the maximum height of the cylinder was 29.8 mm, with a minimum of 12.2 mm. The maximum diameter was 33.8 mm, with a minimum of 7.7 mm.

In the assessment of cement distribution, structures with thicknesses ranging from 400 to 700 microns were of great interest from the practitioner's perspective, especially due to the cloud‐like diffusion observed at the end of the needle (visualized on the CT slices and fluoroscopy in Table 4). The practitioner also reported that structures with a thickness exceeding 700 microns exhibited an excessive retention of cement on the upper surface of the cube. In terms of haptic feedback, the practitioner indicated two structures that were reproducing the haptic feedback during needle insertion into healthy cancellous bone: the structures with a thicknesses of 500 and 600 microns. In comparison, structures with a thickness of over 600 microns were reported to be stiffer than healthy bone, while structures with a thickness of 400 microns was reported to be softer than healthy bone. All stochastic lattices exhibited a weight difference of less than 5%.

#### 3.1.3 Lattice density

In the final series of tests varying the density of lattices, the maximum quantity of injected cement was 6.0 mL, while the minimum quantity was 2.8 mL. Consequently, the maximum height of the cylinder was 30.0 mm, with a minimum of 21.1 mm. Likewise, the maximum diameter was 31.4 mm, while a minimum of 22.7 mm.

In the evaluation of cement distribution, the structure with a density of 15% offered the most accurate representation of the cement diffusion in healthy cancellous bone, based on the practitioner's assessment. The clinician reported that the structure with a density of 10% and 5% could be representative of the cement diffusion in metastatic bone, with varying degrees of osteolysis. In terms of haptic feedback, the practitioner indicated that the 15% density structure was able to reproduce the haptic feedback when a needle was inserted into healthy cancellous bone. The structure with 10% density or below were observed to be less rigid than healthy bone. All stochastic lattices exhibited a weight difference of less than 5%.

### 3.2 Patient specific case

**FIGURE 6:**
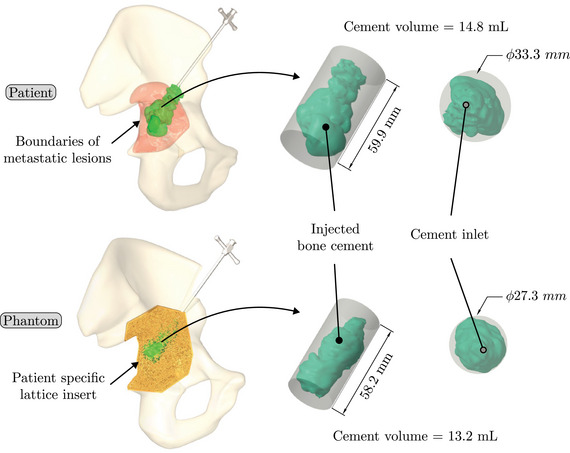
Injected bone cement for the patient and corresponding phantom.

Figure 6 presents the 3D reconstruction of volume of cement injected in the patient and the phantom. The comparison between the patient case and the phantom regarding the diffusion and quantity of cement in the different parts of the bone is reported in Table 7.

**TABLE 7:** Comparative analysis of patient and phantom injection images.

	Patient case	Phantom
Fluoroscopy image	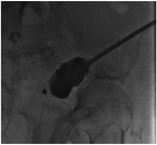	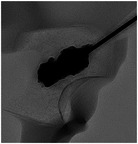
CT scan slice in healthy bone	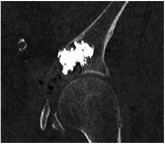	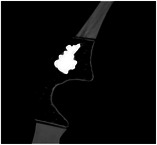
Cement quantity in healthy bone	3.5 mL	1.1 mL
CT scan slice in osteolytic bone	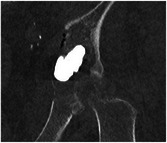	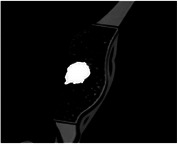
Cement quantity in osteolytic bone	11.3 mL	12.1 mL
Total cement quantity	14.8 mL	13.2 mL
Cylinder [height x diameter]	59.9×14.8 mm	58.2×13.2 mm

The difference between the insert's weight measured in CAD and the actual weight of the insert in the patient case averaged 1.3%, with a standard deviation of 0.9%.

The total cost including material, machine and manpower for a reusable phantom and an insert amounted to 48 and €34 respectively. The production cost of the entire system (pelvis with one insert) amounted to €82. Further details on costs were presented in appendix.

## 4 DISCUSSION

### 4.1 Disposable insert

The preliminary objective of this study was to assess the capacity of various lattice structures to replicate the injection of orthopedic cement into bone tissue affected by osteolytic metastases. Three parameters were investigated: the type of lattice, the thickness of the structures, and the density.

The results of this study revealed that stochastic lattice structures exhibited the closest simulation of cement diffusion within cancellous bone. These types of lattice structures result in a cloud‐like diffusion of cement within the insert, which is similar to the diffusion observed in bones. In contrast, the TPMS structures exhibited a more directional diffusion pattern. This behavior can be attributed to the regular, patterned geometry of TPMS, which are generated by the thickening of a set of minimal surfaces. Consequently, the cement can only flow along distinct paths, represented by volumes A and B in the Figure 2.

In terms of inserts quality, the weight difference was approximately 5% for stochastic lattices and over 10% for TPMS structures. This discrepancy is likely due to the capacity of the structure to remove unpolymerized resin during postprocessing. TPMS structures have partially closed cells compared to stochastic lattices, which could explain this higher difference. Nevertheless, all structures demonstrate a standard deviation of less than 2%, indicating that despite the introduction of weight variations, these discrepancies are consistent and reproducible.

### 4.2 Phantom pelvis

Based on the lattice analysis, a methodology for designing a phantom from patient data was proposed and evaluated.

Different structures have been assigned to the areas of healthy and pathological bone identified on the patient images. The structures replicate the haptic feedback during needle insertion and cement injection in healthy and pathological bone. The phantom pelvis can be used under fluoroscopic guidance (for needle placement and injection) and computed tomography (for needle placement). The methodology presented in Section 2.3.1 allows for the development of other metastasis scenarios, such as metastatic involvement in the iliac wing or pubic rami. To our knowledge, this is the first developed phantom for the training of practitioners for pelvic cementoplasty. As a result, it is challenging to assess the economic impact of our solution. Nevertheless, a total production cost of €82 appears to be a competitive offer compared to other anatomical pelvis models, such as the Sawbones models, which are considered as the gold standard in biomechanical testing.

We acknowledge certain limitations to the developed phantom. The area corresponding to the trabecular bone is confined to the insert and does not include all pelvic bones, due to the selected design strategy. Furthermore, the phantom does not include the soft tissue surrounding the pelvis, which affects both the penetration of the needle into the bone and the flow of cement (in the case of extraosseous leakage, for example). Despite its capacity to reproduce cement diffusion, the designed cancellous bone insert differs significantly from the dimensions of real bone reported in the literature. Moreover, while trabecular bone is typically filled with bone marrow, the insert used in this study was hollow. Our approach aimed to represent pathological bone with a progressively less dense structure, which may however not represent accurately all types of metastases that exhibit various patterns of bone destruction in the clinical practice. Finally, despite the large number of studied structures, only one expert practitioner evaluated our data.

Our future research should aim to address these challenges and explore new avenues to enhance the understanding of pelvic cementoplasty. In particular, we aim to extend the scope of the phantom by addressing additional challenges associated with the injection of orthopedic cement into the pelvis. One of the primary concerns is the occurrence of leaks, which our phantom may simulate, especially extraosseous leaks that can lead to complications involving nerves or joints.^1^, ^31^ A further emerging application of pelvic cementoplasty that our phantom has the potential to address is the combination of screws with orthopedic cement, a technique used for the stabilization of impending/pathological fractures. Various types of constructs have been proposed in the preclinical and clinical literature but without standardization amongst publications.^5^, ^6^ Ultimately, these advancements will contribute to a more comprehensive understanding of pelvic cementoplasty, particularly by training practitioners to perform various procedure, thereby improving the quality of bone stabilization and patient mobility.

## 5 CONCLUSION

Despite the popularity of anatomical models in medical training, there is currently no pelvic bone phantom specifically designed for practicing cement injection. The first part of the study was dedicated to the analysis of different lattice structures by varying three properties (type, thickness, and density) to determine the structures that most accurately reproduce haptic feedback during needle insertion and cement diffusion in diseased and healthy cancellous bone. The results of this analysis were then applied in the design and manufacture of a phantom dedicated to cementoplasty in the pelvic bone. The current pelvic bone phantoms offer the following capabilities: (i) adjusting the orientation and insertion of needles into the pathological area, providing the haptic feedback of needle penetration in healthy and osteolytic metastasis; (ii) adapting the shape and dimensions of disposable inserts dedicated to specific areas; and (iii) reproducing, under fluoroscopic and CT scan guidance, the diffusion of cement within healthy and diseased cancellous bone.

## CONFLICT OF INTEREST STATEMENT

The authors have no conflicts to disclose.

## References

[1] Park JW , Lim HJ , Kang HG , Kim JH , Kim HS . Percutaneous cementoplasty for the pelvis in bone metastasis: 12‐year experience. Ann Surg Oncol. 2022;29:1413‐1422. 34448054 10.1245/s10434-021-10640-8 PMC8390074

[2] Garnon J . Assistance to the injection of a large volume of orthopedic bone cement . Phd thesis. University of Strasbourg; 2020. https://theses.fr/2020STRAD036

[3] Yevich S , Tselikas L , Gravel G , Baère Td , Deschamps F . Percutaneous cement injection for the Palliative treatment of Osseous metastases: a technical review. Semin Intervent Radiol. 2018;35(04):268‐280. 30402010 10.1055/s-0038-1673418 PMC6218257

[4] Garnon J , Jennings JW , Meylheuc L , et al. Biomechanics of the Osseous pelvis and its implication for consolidative treatments in interventional oncology. Cardiovasc Intervent Radiol. 2020;43(11):1589‐1599. 32851425 10.1007/s00270-020-02624-0

[5] Dassa M , Roux C , Tselikas L , et al. Image‐guided percutaneous fixation with internal cemented screws of impending femoral neck pathologic fractures in patients with metastatic cancer: safety, efficacy, and durability. Radiology. 2020;297(3):721‐729. 33021894 10.1148/radiol.2020201341

[6] Roux C , Tselikas L , Yevich S , et al. Fluoroscopy and cone‐beam CT–guided fixation by internal cemented screw for pathologic pelvic fractures. Radiology. 2019;290(2):418‐425. 30422090 10.1148/radiol.2018181105

[7] Moser TP , Onate M , Achour K , Freire V . Cementoplasty of pelvic bone metastases: systematic assessment of lesion filling and other factors that could affect the clinical outcomes. Skeletal Radiol. 2019;48(9):1345‐1355. 30712119 10.1007/s00256-019-3156-0

[8] Garnon J , Meylheuc L , De Marini P , et al. Cement plug fragmentation following percutaneous cementoplasty of the bony pelvis: is it a frequent finding in clinical practice?. Cardiovasc Intervent Radiol. 2021;44(3):421‐427. 33241471 10.1007/s00270-020-02715-y

[9] Tenewitz C , Le RT, Hernandez M , Baig S , Meyer TE . Systematic review of three‐dimensional printing for simulation training of interventional radiology trainees. 3D Print Med. 2021;7(1):10. 33881672 10.1186/s41205-021-00102-y PMC8059217

[10] Stefan P , Pfandler M , Lazarovici M , et al. Three‐dimensional–printed computed tomography–based bone models for spine surgery simulation. Simul Healthc. 2020;15(1):61‐66. 32028448 10.1097/SIH.0000000000000417

[11] Filippou V , Tsoumpas C . Recent advances on the development of phantoms using 3D printing for imaging with CT, MRI, PET, SPECT, and ultrasound. Med Phys. 2018;45(9):e740‐e760. 29933508 10.1002/mp.13058 PMC6849595

[12] Rai R , Manton D , Jameson MG , et al. 3D printed phantoms mimicking cortical bone for the assessment of ultrashort echo time magnetic resonance imaging. Med Phys. 2018;45(2):758‐766. 29237232 10.1002/mp.12727

[13] Bortman J , Baribeau Y , Jeganathan J , et al. Improving clinical proficiency using a 3‐dimensionally printed and patient‐specific thoracic spine model as a haptic task trainer. Reg Anesth Pain Med. 2018;43(8):819‐824. 29894394 10.1097/AAP.0000000000000821

[14] Mei K , Pasyar P , Geagan M , et al. Design and fabrication of 3D‐printed patient‐specific soft tissue and bone phantoms for CT imaging. Sci Rep. 2023;13(1):17495. 37840044 10.1038/s41598-023-44602-9 PMC10577126

[15] Hatamikia S , Kronreif G , Unger A , et al. 3D printed patient‐specific thorax phantom with realistic heterogenous bone radiopacity using filament printer technology. Z Med Phys. 2022;32(4):438‐452. 35221154 10.1016/j.zemedi.2022.02.001 PMC9948829

[16] Tatarinov A , Panov V . Physical models of cortical bone conditions, fabricated by a 3D printer to test for sensitivity of axial transmission technique. In: 2015 6th European Symposium on Ultrasonic Characterization of Bone . IEEE; 2015:1‐4.

[17] Burns J , Mansouri M , Wake N . Chapter 10 ‐ 3D printing in radiology education. 3D Printing for the Radiologist. 2022:117‐129.

[18] Hao J , Nangunoori R , Wu YY , et al. Material characterization and selection for 3D‐printed spine models. 3D Print Med. 2018;4(1):8. 30649649 10.1186/s41205-018-0032-9 PMC6195498

[19] Burkhard M , Fürnstahl P , Farshad M . Three‐dimensionally printed vertebrae with different bone densities for surgical training. Eur Spine J. 2019;28(4):798‐806. 30511245 10.1007/s00586-018-5847-y

[20] Mitsouras D , Liacouras P , Imanzadeh A , et al. Medical 3D Printing for the Radiologist. Radiographics. 2015;35(7):1965‐1988. 26562233 10.1148/rg.2015140320 PMC4671424

[21] Fedorov A , Beichel R , Kalpathy‐Cramer J , et al. 3D Slicer as an image computing platform for the quantitative imaging network. Magn Reson Imaging. 2012;30(9):1323‐1341. 22770690 10.1016/j.mri.2012.05.001 PMC3466397

[22] Creo CAD Software — PTC. https://www.ptc.com/en/products/creo

[23] Rengier F , Mehndiratta A , Tengg‐Kobligk vH , et al. 3D printing based on imaging data: review of medical applications. Int J Comput Assist Radiol Surg. 2010;5(4):335‐341. 20467825 10.1007/s11548-010-0476-x

[24] Lea WB , Neilson JC , King DM , Tutton SM . Minimally invasive stabilization using screws and cement for pelvic metastases: technical considerations for the pelvic “screw and glue” technique. Semin Intervent Radiol. 2019;36(3):229‐240. 31435131 10.1055/s-0039-1693982 PMC6699961

[25] Shi J , Zhu L , Li L , Li Z , Yang J , Wang X . A TPMS‐based method for modeling porous scaffolds for bionic bone tissue engineering. Sci Rep 2018;8(1):7395. 29743648 10.1038/s41598-018-25750-9 PMC5943328

[26] Chen H , Han Q , Wang C , Liu Y , Chen B , Wang J . Porous scaffold design for additive manufacturing in orthopedics: a review. Front Bioeng Biotechnol. 2020;8:609. 32626698 10.3389/fbioe.2020.00609 PMC7311579

[27] Arabnejad S , Johnston RB , Pura JA , Singh B , Tanzer M , Pasini D . High‐strength porous biomaterials for bone replacement: a strategy to assess the interplay between cell morphology, mechanical properties, bone ingrowth and manufacturing constraints. Acta Biomater. 2016;30:345‐356. 26523335 10.1016/j.actbio.2015.10.048

[28] Liu S , Chen J , Chen T , Zeng Y . Fabrication of trabecular‐like beta‐tricalcium phosphate biomimetic scaffolds for bone tissue engineering. Ceram Int. 2021;47(9):13187‐13198.

[29] Bailey S , Hackney D , Vashishth D , Alkalay RN . The effects of metastatic lesion on the structural determinants of bone: current clinical and experimental approaches. Bone. 2020;138:115159. 31759204 10.1016/j.bone.2019.115159 PMC7531290

[30] Nazarian A , Stechow vD , Zurakowski D , Müller R , Snyder BD . Bone volume fraction explains the variation in strength and stiffness of cancellous bone affected by metastatic cancer and osteoporosis. Calcif Tissue Int. 2008;83(6):368‐379. doi:10.1007/s00223-008-9174-x 18946628

[31] Miranda MO , Moser TP . A practical guide for planning pelvic bone percutaneous interventions (biopsy, tumour ablation and cementoplasty). Insights Imaging. 2018;9(3):275‐285. 29564836 10.1007/s13244-018-0600-y PMC5991000

[32] Bohl MA , Mauria R , Zhou JJ , et al. The Barrow biomimetic spine: face, content, and construct validity of a 3D‐printed spine model for freehand and minimally invasive pedicle screw insertion. Global Spine J. 2019;9(6):635‐641. 31448198 10.1177/2192568218824080 PMC6693063

[33] Li Y , Li Z , Ammanuel S , Gillan D , Shah V . Efficacy of using a 3D printed lumbosacral spine phantom in improving trainee proficiency and confidence in CT‐guided spine procedures. 3D Print Med. 2018;4(1):7. 30649653 10.1186/s41205-018-0031-x PMC6179970

